# Boosting Magnetoelectric Effect in Polymer-Based Nanocomposites

**DOI:** 10.3390/nano11051154

**Published:** 2021-04-28

**Authors:** Alexander Omelyanchik, Valentina Antipova, Christina Gritsenko, Valeria Kolesnikova, Dmitry Murzin, Yilin Han, Andrei V. Turutin, Ilya V. Kubasov, Alexander M. Kislyuk, Tatiana S. Ilina, Dmitry A. Kiselev, Marina I. Voronova, Mikhail D. Malinkovich, Yuriy N. Parkhomenko, Maxim Silibin, Elena N. Kozlova, Davide Peddis, Kateryna Levada, Liudmila Makarova, Abdulkarim Amirov, Valeria Rodionova

**Affiliations:** 1 REC Smart Materials and Biomedical Applications, Immanuel Kant Baltic Federal University, 236041 Kaliningrad, Russia; asomelyanchik@kantiana.ru (A.O.); vantipova1@kantiana.ru (V.A.); christina.byrka@gmail.com (C.G.); vgkolesnikova1@kantiana.ru (V.K.); dvmurzin@yandex.ru (D.M.); elevada@kantiana.ru (K.L.); la.loginova@physics.msu.ru (L.M.); 2 Department of Chemistry and Industrial Chemistry (DCIC), University of Genova, 16146 Genova, Italy; davide.peddis@unige.it; 3 Biomedical Centre, Department of Neuroscience, Uppsala University, 751 24 Uppsala, Sweden; yilin.han@neuro.uu.se (Y.H.); elena.kozlova@neuro.uu.se (E.N.K.); 4 Laboratory of Physics of Oxide Ferroelectrics and Department of Materials Science of Semiconductors and Dielectrics, National University of Science and Technology MISiS, 119049 Moscow, Russia; aturutin92@gmail.com (A.V.T.); kubasov.ilya@gmail.com (I.V.K.); akislyuk94@gmail.com (A.M.K.); ilina.tatina@gmail.com (T.S.I.); dm.kiselev@gmail.com (D.A.K.); mvoron@bk.ru (M.I.V.); malinkovich@yandex.ru (M.D.M.); parkh@rambler.ru (Y.N.P.); 5 Department of Physics and I3N, University of Aveiro, 3810-193 Aveiro, Portugal; 6 Institute of Advanced Materials and Technologies, National Research University of Electronic Technology “MIET”, 124498 Moscow, Russia; sil_m@mail.ru; 7 Institute for Bionic Technologies and Engineering, I.M. Sechenov First Moscow State Medical University, 119991 Moscow, Russia; 8 Scientific-Manufacturing Complex “Technological Centre” Shokin Square, House 1, Bld. 7, Zelenograd, 124498 Moscow, Russia; 9 Institute of Structure of Matter–CNR, Monterotondo Stazione, 00016 Rome, Italy; 10 Faculty of Physics, Lomonosov Moscow State University, 1-2 Leninskie Gory, 119234 Moscow, Russia; 11 Amirkhanov Institute of Physics of Dagestan Federal Research Center, Russian Academy of Sciences, 367003 Makhachkala, Russia

**Keywords:** multiferroics, magnetoelectric effect, nanoparticles, cobalt ferrite, barium titanate, PVDF, PVDF-TrFE

## Abstract

Polymer-based magnetoelectric composite materials have attracted a lot of attention due to their high potential in various types of applications as magnetic field sensors, energy harvesting, and biomedical devices. Current researches are focused on the increase in the efficiency of magnetoelectric transformation. In this work, a new strategy of arrangement of clusters of magnetic nanoparticles by an external magnetic field in PVDF and PFVD-TrFE matrixes is proposed to increase the voltage coefficient (α_ME_) of the magnetoelectric effect. Another strategy is the use of 3-component composites through the inclusion of piezoelectric BaTiO_3_ particles. Developed strategies allow us to increase the α_ME_ value from ~5 mV/cm·Oe for the composite of randomly distributed CoFe_2_O_4_ nanoparticles in PVDF matrix to ~18.5 mV/cm·Oe for a composite of magnetic particles in PVDF-TrFE matrix with 5%wt of piezoelectric particles. The applicability of such materials as bioactive surface is demonstrated on neural crest stem cell cultures.

## 1. Introduction

Multiferroics are a class of material where magnetism and ferroelectricity coexist in coupling and synergy. The development of new composite multiferroic materials with better properties than in single-phase multiferroics, having the interrelated piezoelectric and ferromagnetic properties once again take a lot of attention [1,2,3]. Coupled electrical polarization and magnetization give rise to their mutual control. For example, the direct magnetoelectric (ME) effect is the magnetically tunable polarization, change of the value or direction of electrical polarization under the applied magnetic field. Those unique properties are advance for the application of ME composites in energy transfer/harvesting [4,5,6], magnetic field sensors [5,7,8] and biomagnetic field sensors [9,10].

Magnetorheological smart materials are a class of composite materials having both rheological and magnetic properties [11]. This kind of material is usually composed of ferro (i-) magnetic micro- or nanofiller and elastic polymer matrix [12]. One of the advantages of the elastic polymer composites is that they can be easily shaped for a specific application, for instance, via using a 3d-printer [13]. It gives rise to interest in the utilization of magnetorheological composites in different applications as mechanical manipulators, actuators, tunable dampers, as well as soft robots, etc. [14].

If the above properties (multiferroics and magnetorheological) are met in one continuity, these materials will merge attributes and advantages from both families. An interesting example is represented by the magnetoelectric polymeric composites—materials consisting of magnetic/magnetostrictive filler (e.g., magnetic nanoparticles (NPs)) and piezopolymer matrix or polymer-bonded composites of ferroelectric and magnetic particles [15,16]. In this class of materials, ME coupling occurs through strain interactions (elastic coupling) of magnetic filler and piezoelectric particles or matrix [12,17,18,19]. As we know, the magnitude of the magnetoelectric effect in elastic polymer-bonded composites is by an order of magnitude larger (~700 mV/cm·Oe [20]) compared to the composites based on a piezoelectric polymeric matrix and magnetic nanoparticles (~40 mV/cm·Oe [15,18]). This fact can be explained by a different mechanism of coupling: in bonded composites, elastic coupling was explained by the mutual movement of two kinds of particles (magnetic in a magnetic field and ferroelectric in an electric field, respectively) [21], while in the piezopolymeric matrix is due to magnetostriction of magnetic fillers [15]. The highest value of ME effect in polymer-bonded composites however was achieved in composites of micron-sized particles of lead zirconate titanate (PZT) and neodymium iron boron (NdFeB) [20], which do not meet the requirements of biocompatibility. The goal of this work is to keep the relatively high biocompatibility of composites based on a piezoelectric polymeric matrix, to decrease the amount of inorganic inclusions and to achieve a high value of ME effect at the same time.

Despite the magnetoelectric effect in polymeric nanocomposites (NCs) is still smaller than in ceramic or laminar structures, they have advantages in simple fabrication, flexibility, and easy shaping [15]. Additionally, polymeric interfaces can show good biocompatibility, which together with multiferroic properties make them a unique tool for a set of bioapplications (e.g., cultivation surfaces with remotely controlled electric surface charge and mechanical stresses by applying an external magnetic field [22,23]). Application of both stimuli—charge and mechanical stress—may promote cell responses such as a controlled differentiation of stem cells. The physical stimulation of stem cell differentiation can replace the biochemical methods that are being used at the current time in stem cell-based therapy of neurodegenerative disorders [24]. Differentiation of stem cells into osteocytes [25], cardiomyocytes [26], and neural cells [27], initiated by electrical and mechanical stimulus has been studied. Neural cells are more sensitive to electrical stimulation because of their electric activities. Electrical stimulus induced by piezoelectric polymers leads to targeted axonal growth, inducing directed cell migration and promoting neurogenesis [24]. The first step of the investigation of physical factors’ effects on neuronal stem cell differentiation is their cultivation on piezoelectric polymers that are the tests for biocompatibility with followed targeted differentiation. The biocompatibility of PVDF was demonstrated earlier on neuronal stem cells, isolated at the later stage of embryonic development [28]. Using the neural stem cells isolated in the early embryonic period can increase the ability to direct their differentiation for future applications [29]. The biocompatibility effect of PVDF on the neural stem cells isolated in the early embryonic period should be studied additionally. In vitro cell or organ growing for further transplantation is an attractive stem cell-based therapy for the treatment of neurodegenerative disorders such as Parkinson’s disease, Huntington’s disease, Alzheimer’s, amyotrophic lateral sclerosis [30], spinal cord injury [28], and brain damage [31].

In this work, we prepared NCs based on two types of polymers, poly(vinylidene fluoride) (PVDF) and its copolymers with trifluoroethylene (PVDF-TrFE) [32]. NCs based on PVDF-TrFE demonstrated a higher magnetoelectric performance and thus were chosen for further experiments. The highly crystalline CoFe_2_O_4_ NPs were prepared via a sol-gel auto-combustion method [33] and they were used for the preparation of rheological magnetoelectric materials [19]. Further, new strategies to increase magnetoelectric response were involved: (i) application of magnetic field during crystallization of polymer to align clusters of magnetic NPs and (ii) creation of 3-component composite with ferroelectric BaTiO_3_ particles. We tested the piezoelectric polymers for future application as biointerfaces for activation and targeted differentiation of neuronal stem cells: neuronal stem cells isolated at the early embryonic stage cultivated on PVDF-based surface were able to proliferate and differentiate into main types of neural cells (neurons and glial cells).

## 2. Materials and Methods

### 2.1. Synthesis of CoFe_2_O_4_ (CFO), Zn_0.25_Co_0.75_Fe_2_O_4_ (ZCFO) and BaTiO_3_ (BTO) Particles

Samples of CoFe_2_O_4_ NPs were prepared by the self-combustion method described in detail elsewhere [33]. The Fe(NO_3_)_3_·9H_2_O (Carlo Erba Reagenti SpA, Cornaredo, Italy), Co(NO_3_)_2_·6H_2_O (Scharlab S.L, Barcelona, Spain), citric acid (Scharlab S.L., Barcelona, Spain), and of 30% ammonia solution (Carlo Erba Reagenti SpA, Cornaredo, Italy) were used without further purification. In this process, 1-molar iron and cobalt nitrate aqueous solutions in a 2:1 ratio, respectively, and citric acid with 1:1 molar ratio of metals to citric acid were prepared. The pH level was adjusted to the value of ~7 by dropwise addition of aqueous ammonia. Obtained sol was placed on a hotplate at 150 °C to form a gel for 2 h. The gels underwent successively a thermal treatment at 300 °C for 15 min, where the auto-combustion reaction took place. Additionally, the Zn substituted cobalt ferrite (Zn_0.25_Co_0.75_Fe_2_O_4_, ZCFO) NPs were prepared with the same sol-gel auto-combustion method. A more detailed characterization of ZCFO NPs used here is reported earlier [34].

BaTiO_3_ (BTO) particles were prepared by the solid-phase reaction method, followed by sintering using conventional ceramic technology described in detail elsewhere [35]. Briefly, BaCO_3_ and TiO_2_ powder with a purity of at least 99.95% were used as precursors. Then, BaTiO_3_ particles were prepared by solid-state reaction method in two stages: at T_1_ = 1150 °C during time τ_1_ = 4 h (1st stage) and T_2_ = 1170 °C in during τ_2_ = 4 h (2nd stage).

### 2.2. Fabrication of Magnetoelectric Nanocomposites (NCs)

For composite fabrication, the two different types of polymers, poly(vinylidene fluoride) (PVDF) and its copolymer with trifluoroethylene (PVDF-TrFE) were used as a polymer matrix. For the preparation of the polymer-precursor solution, PVDF (Alfa Aesar, Kandel, Germany) or PVDF-TrFE 55/45 (Piezotech, King of Prussia, PA, USA) granules were dissolved in dimethylformamide (DMF) (Sigma-Aldrich, Darmstadt, Germany) at 40 °C followed by mixing until complete dissolution of polymer granules. The concentrations were about 1:4 in weight ratio for PVDF/DFM and 1:6 for PVDF-TrFE/DMF solution. The dissolution time was about 45 min for PVDF and 90 min for PVDF-TrFE. The total concentrations of PVDF/DMF and PVDF-TrFE/DMF were 1:8 and 1:12, respectively, since at the next step an additional amount of DMF was introduced together with NPs.

Nanocomposites of NPs embedded in the piezoelectric polymer matrix were fabricated by the solvent evaporation method assisted by a *doctor blade* technique [36]. The so-called doctor blade or blade coating method is one of the simple methods for lab-scale production of thin polymer composites. In this method, the polymer solution is placed on the substrate in front of the moving blade and is smoothed out by it. The thickness of the layer is controlled by adjusting a gap between the knife (blade) and substrate. The thickness of the final evaporated layer depends on the gap between knife and substrate, speed of coating, the temperature of the substrate and physical properties of solution (viscosity, density, etc.). The technological protocol of composite fabrication is strongly dependent on the type of polymer, fillers, and type of solvent. The CFO or ZCFO NPs were ground, mixed with the second part of DMF solvent and dispersed in preliminary prepared polymer-precursor solutions in an ultrasonic bath for 2 h. The mixing of fillers in DMF solutions was applied to decrease the particle agglomerations and their more homogeneous distribution in polymer solutions. In the next set of samples, in the system demonstrated higher magnetoelectric properties (oriented CFO/PVDF-TRfE), 5% and 10% weight content of BaTiO_3_ (BTO) particles was added at the same step as CFO particles.

**Figure 1:**
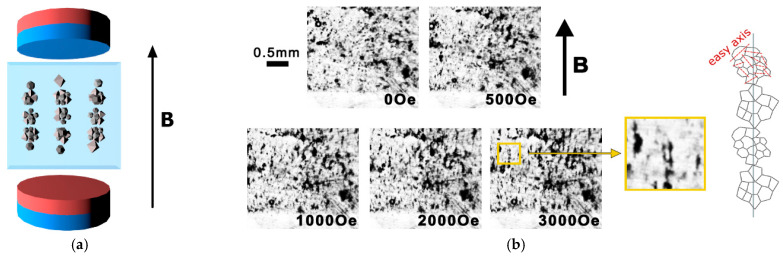
(**a**) Illustration of the alignment of CFO NPs in PVDF polymer in a magnetic field; (**b**) optical image of the formation of ordered chains of CFO NPs clusters in the liquid precursor of PVDF-TrFE polymer under external magnetic fields of different inductions. The sketch represents a structure of the chain as an elongated assembly of NPs clusters with the random distribution of easy axes of individual particles inside each cluster (red lines).

The solution of particles and polymers precursor was spread on a clean glass substrate using a coating blade at a fixed distance between the substrates. The solvent was evaporated by heating the composites in an oven at 75 °C for 15 min. Then, for the fabrication of the ordered samples, this protocol was modified as follows: clusters of magnetic NPs were aligned in the magnetic field before evaporation of the polymer’s solvent. The magnetic field was applied in-plane of the dish with the precursor solution during evaporation (Figure 1a). After evaporation, the particles were immobilized in a polymer matrix, when the magnetic field was removed, aligned samples were obtained.

Figure 1b shows the alignment process of clusters composed of CFO NPs into chains in a gradually increasing magnetic field up to 3 kOe. Electromagnets of the magnetometer (7400 System VSM; Lake Shore Cryotronics Inc., Westerville, OH, USA) were used to generate a homogeneous magnetic field (±0.1%) in a volume 10 mm^3^, which is bigger than the volume of the samples (typical shape of sample is a square with edges of 4 mm and thickness in the range of 30–60 μm). After switching of the magnetic field, optical images were obtained with a 5.3 MP monochrome camera PixeLINK PL-D725MU-T (Edmund Optics Inc., Barrington, NJ, USA) placed between two coils of the electromagnet. At a field of about ~500 Oe, clusters of particles start to move, forming aligned structures. At a field of about ~3 kOe, those structures achieved the final state and the further increase of field does not change the shape of the clusters’ chains. The clusters of CFO NPs in PVDF-TrFE-based solutions showed better alignment in the magnetic field, because of higher viscosity and lower time of drying in comparison with PVDF-based (Figure S5). Moreover, the difference between the two polymers was in the structure of the surface. According to the atomic force microscopy (AFM), the pore size was 30 ± 12 nm in PVDF-TrFE-based and 100 ± 64 nm in PVDF-based NCs (see explanation in SI, Figure S6). The same experiment of registration of movement and reorganization of particle aggregations, performed on samples after evaporation, showed that the particles were rigidly fixed in the polymer matrix (no displacement was detected within experimental error).

Finally, magnetoelectric NCs were obtained by detaching the glass substrate. All samples were prepared with 15% weight content (wt.%) of CFO or ZCFO NPs, because according to literature data around the enhanced formation of ferroelectric β- and γ-phases of PVDF polymers is expected [37].

All samples were poled using direct contact poling in a custom-designed chamber for 40 min at 40 °C [38]. The chamber was constructed as the adiabatic camera from thermo-insulated material (polystyrene foam) and equipped with a thermoregulation system. The maximal poled electric field was 50 MV/m.

### 2.3. Structural and Magnetic Characterization

The X-ray diffraction (XRD) studies were performed with a DaVinci2 diffractometer (Bruker, MA, USA) using Cu Kα (λ = 1.54056 Å) in the 2θ geometry in a range of 10–70 degrees. The average size of crystallites d_XRD_ was calculated for (440) peak with Scherrer’s equation:

d_XRD_ = 0.94·λ/(B·cos θ) (1)

where B is full width at half maximum (FWHM) and θ is a position of XRD reflections.

The size distribution of NPs was investigated by using an S-5500 Transmission Electron Microscopy (TEM; Hitachi, Japan).

The magnetic properties were studied with a vibrating sample magnetometer 7400 System (VSM; Lake Shore Cryotronics Inc., Westerville, OH, USA) in the magnetic field up to 1.1 T at room temperature (295 K). Since the maximal acquired field was not sufficient to fully saturate the sample, the value of saturation magnetization was extrapolated with the fitting of the high-field region using the Law of Approach to Saturation (LAS):

M(H) = M_S_(1 − A/H − B/H^2^) (2)

where A and B are fitting parameters [39]. The Equation (2) was applied previously to estimate M_S_ in different ferrite nanoparticles [34,40].

A deeper investigation of the magnetic properties was conducted by FORC analysis (first-order reversal curve [41,42]). To measure the FORC, the sample was first saturated, then the applied field was decreased to the value of the return field (H_r_). The curve measured from the H_r_ to the saturation field is a single FORC. The cycle of at least 100 repetitions by decreasing the value of the H_r_ was recorded. The measurement of such curves provides information from different paths of magnetization and interaction fields for all the phases that contribute to the hysteresis loop. FORC method was recently applied to study the magnetomechanical properties of magnetic elastomers (intrinsic magnetic hysteresis of magnetic filler and mechanical compliance of the matrix) [43]. The FORC diagram interpretation is based on its comparison with the Classical Preisach Model of hysteresis [44]. In this model, the hysteron is a mathematical operator that acts on the magnetic field and produces a square hysteresis loop characterized by a coercive field H_c_ (half-width) and an interaction field H_u_ (horizontal bias) [45]. Each magnetic phase in the material could be described by a single hysteron, while a set of hysterons will describe the macroscopic hysteresis cycle of the entire sample. The hysteron’s distribution ρ(H_c_, H_u_) is represented on the two-dimensional Preisach plane with H_c_ and H_u_ axis profiles. By comparing the H_c_ and H_u_ axis profiles the information about the magnetic interactions in the system can be obtained [46]. The FORC-curves were obtained via 7400 VSM FORC Utility (Lake Shore Cryotronics Inc., Westerville, OH, USA).

### 2.4. Magnetoelectric Properties

**Figure 2:**
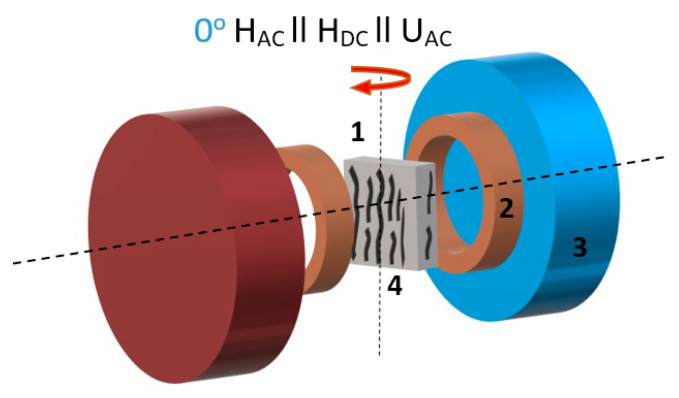
Scheme of the experiment for direct magnetoelectric (ME) measurements (**1**—sample, **2**—Helmholtz coils, **3**—DC magnetic field source, **4**—aligned chains of particle clusters); the red arrow indicates the direction in which sample was rotated.

The ME studies were carried out using a custom-designed setup for measuring the magnetoelectric voltage Δ*V* with a lock-in amplifier (Model SR830, Stanford Research, Sunnyvale, CA, USA) at frequencies of 1 Hz–100 kHz. The input impedance of the lock-in amplifier is 10 MOhm. The ME coefficient α_ME_ was defined using the following equation:

(3) $$\alpha_{\mathrm{ME}}=\frac{\Delta V}{b\;\Delta H}$$

where Δ*V* is the amplitude of the induced ME voltage, *b* is the thickness of the sample, and Δ*H* is the amplitude of the AC field H_AC_. The accuracy of ME signal measurements was less than 1%. The amplitude of H_AC_ was about 10 Oe and the DC field was varied up to 10 kOe. The H_AC_ and H_DC_ fields were applied across the plane of the sample, that is H_AC_‖H_DC_‖Δ*V* (Figure 2). The Helmholtz coils were used for the generation of AC field, the DC bias field was applied using an adjustable Halbach type magnet system (AMT & C LLC, Troitsk, Russia). Electric contacts were made by the coating of aluminum foils on the larger surface of composite films, thus, the ME coefficient was measured in α_33_ mode.

### 2.5. Magnetic and Piezoresponse Force Microscopy

Piezoresponse force microscopy (PFM) and local polarization switching spectroscopy measurements were carried out with MFP-3D (Asylum Research, Goleta, CA, USA) commercial scanning probe microscope using the CSG30/Pt (Tipsnano, Tallinn, Estonia) conductive probe with the spring constant of 0.6 N/m. The PFM out-of-plane images were scanned in the single frequency PFM mode at 3 V and a frequency of ~7 kHz. An alternating current (AC) voltage (3 V) was superimposed onto a triangular square-stepping wave (f = 0.5 Hz, with writing and reading times 25 ms, and bias window up to ±150 V) during the remnant piezoelectric hysteresis loops measurements. PFM images were also measured with applying DC magnetic field. The magnetic field (B_ext._ = 1.4 kOe) was applied perpendicular to the plane of the samples. To estimate the effective d_33_ piezoelectric constants, the deflections and vibration sensitivity of the cantilever alignment were calibrated by GetReal procedure using the IgorPro software. For the quantification of switching and piezoelectric coefficient of samples, a dual AC resonance tracking piezoresponse force microscopy (DART-PFM) was employed, which allowed us to probe the piezoresponse that originated within a single domain with a spatial resolution up to submicrometers [47]. DART-PFM is comparatively a reliable technology to probe the piezoresponse from thin polymer samples, because it uses dual AC resonance tracking to quantify the shift of resonance to avoid the noise effects of the surface height topography and suppress the contributions from electrostatic effects [48].

Besides, effective piezoelectric coefficient d_33_(Voltage) hysteresis loops were investigated for further understanding of the magnetic field influence on piezoelectric response. The hysteresis loops (PFM Amplitude (pm) and PFM phase) were acquired using the simple harmonic oscillator (SHO) fit with Asylum Research software to exclude the magnification effect of Q factor of the contact resonance. Effective longitudinal piezoelectric response (“effective d_33_”) was calculated by Equation (4):

d_33_ (pm/V) = (PFM Amplitude (pm) × cos (PFM Phase))/Applied AC voltage (V). (4)

Magnetic force microscopy (MFM) images were obtained using the ASYMFM HC magnetic probe (Asylum Research, Goleta, CA, USA). For MFM scans commercial cantilevers (ASYMFM HC) coated with a magnetic layer of CoPt/FePt (tip apex radius 45 nm; H_C_ > 5 kOe) were utilized. The lift height is 300 nm.

### 2.6. Biological Tests of Polymeric Interfaces

Boundary cap neural crest stem cells (bNCSC) culture is a transient neural crest-derived group of cells located at the dorsal root entry zone. Previous experiments have shown that bNCSCs can differentiate into sensory neurons and glial cells in vitro [29] and in vivo after transplantation [49]. The bNCSC were generated from E11.5 days mouse embryo constitutively expressing red fluorescence signal (RFP) under actin promoter [50]. The bNCSCs were cultured as neurospheres in propagation medium: N2 medium containing bFGF (basic fibroblast growth factor) and EGF (epidermal growth factor) (20 ng/mL, RnD Systems, Minneapolis, MN, USA), and B27 supplement (Gibco, Waltham, MA, USA). bNCSCs were dissociated to single cells with 3PlE and plated on sterilized PVDF substrate (70% alcohol and UV-light for 2 h) covered surface on the bottom of 4-well dishes (D = 16.5 mm). bNCSCs were cultured in proliferation medium (stem cell medium) for 45 min. After that, the medium was replaced with a differentiation medium (DMEM-F12/Neurobasal medium supplemented with N2, B27, 0.1 mM non-essential amino acids and 2 mM sodium pyruvate).

After 72 hours’ incubation time neurospheres on the PVDF substrate were fixed for 15 min with 4% phosphate-buffered paraformaldehyde (PFH, Merk, Darmstadt, Germany) at room temperature (RT) and washed with phosphate buffered saline (PBS, Gibco, Carlsbad, CA, USA) three times for 10 min. Then, the cells were left overnight in PBS at +4 °C. Then in 12 h, cells were washed and incubated in preincubation solution (1% bovine serum albumin (BSA, Thermo Scientific, Waltham, MA, USA), 0.3% Triton X-100 (Invitrogen, Carlsbad, CA, USA), and 0.1% sodium azide (NaN3, Merk, Darmstadt, Germany) in PBS) for 60 min, and incubated with primary antibodies (GFAP; Rabbit, 1:500, Merk, Darmstadt, Germany; III β-Tubulin; Rabbit, 1:500, BioSite, Täby, Sweden) overnight at 4 °C, followed by the appropriate secondary antibodies (Alexa Fluor 488 goat anti-rabbit IgG (H + L; 1:250, Life Technologies, Carlsbad, CA, USA)) for 4 h at RT. Subsequently, the PVDF substrate was washed in PBS, and cells were incubated with Hoechst (1:10,000; Invitrogen, Carlsbad, CA, USA) to label cell nuclei, and then mounted on a glass slide for analysis.

After the immunostaining procedure, PVDF substrate with cells was examined using a fluorescence microscope Eclipse E800 (Nikon, Tokyo, Japan). Image analysis was carried out using ImageJ.

## 3. Results and Discussions

### 3.1. Characterization of CFO and BTO Particles

**Figure 3:**
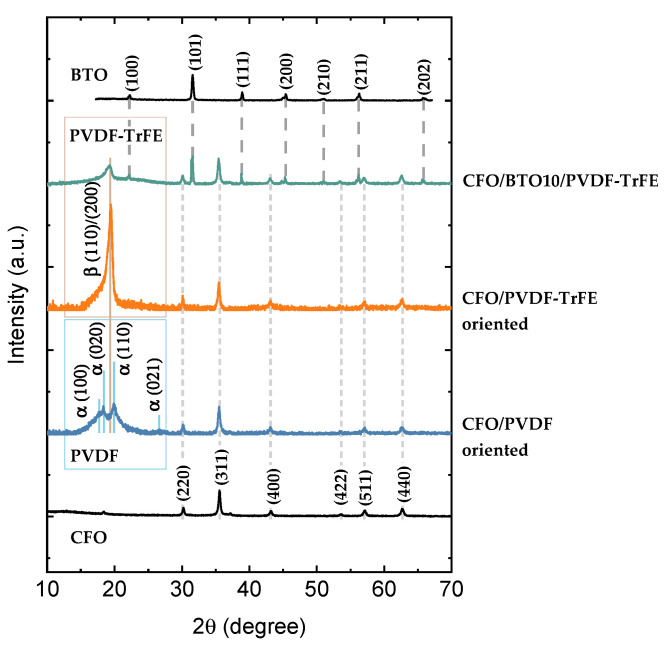
XRD patterns of CFO and BTO nanoparticles, CFO/PVDF, CFO/PVDF-TrFE, and CFO/BTO10/PVDF-TrFE nanocomposites. The Miller indexes specified for pure CFO, BTO particles and PVDF (PVDF-TrFE) polymer are guided to corresponding reflections in composites via dashed lines.

The XRD pattern of the powder CoFe_2_O_4_ (CFO) sample indicates the high crystallinity of nanoparticles without any amorphous content (Figure 3 and more detailed in Figure S1a). The observed reflections were indexed to a cubic spinel lattice according to card No.591-0063 for cobalt ferrite. The size of crystallites calculated by Equation (1) d_XRD_ = 17 ± 2 nm was close to the mean size of the particles observed with TEM microanalysis (Figure S1b, d = 15 ± 1 nm with standard deviation σ = 8 ± 1 nm). This fact indicates the high crystallinity of synthesized NPs. Field dependence of magnetization recorded at 300 K for powder NPs shows hysteretic behavior typical for ferrimagnetic nanoparticles in the blocked state (Figure S2a). The coercivity field (H_C_) was ~1.3 kOe, saturation magnetization (M_S_) was ~66 emu/g, and reduced remanence (M_R_/M_S_) of about 0.44. More detailed characterization of the magnetic and structural properties of particles was already reported elsewhere [34]. Magnetic interparticle interactions were evaluated by measuring the remanence curves and plotting of ΔM-plots (see SI and refs. [51,52] for more details). CFO powder sample shows a negative value of ΔM (Figure S2b) with the maximum intensity of about ~0.1 that suggests that the interparticle dipolar interactions are dominant. The Zn_0.25_Co_0.75_Fe_2_O_4_ (ZCFO) NPs have a similar average crystal size 16 ± 2 nm (Figure S1). Substitution of diamagnetic Zn^2+^ ions in the spinel structure of cobalt ferrite results in a decreased value of magnetic anisotropy (K_CFO_ = 1.6 × 10^6^ erg/cm^3^; K_ZCFO_ = 0.95 × 10^6^ erg/cm^3^ [34]) and a slightly higher value of the saturation magnetization (~74 emu/g) concerning pure CFO sample (Figure S1a). ZCFO powder sample also shows a negative value of ΔM (Figure S2b) of higher magnitude due to a larger saturation magnetization, which led to the stronger dipolar interactions.

The XRD analysis of BaTiO_3_ (BTO) particles indicates the presence of a perovskite tetragonal structure (Figure 3 and more detailed in Figure S3). Positions of main reflections were indexed according to card No.152-5437. Cell parameters were a = 3.995 Å, c = 4.030 Å; V = 64.31Å; c/a = 1.0088, typical for BaTiO_3_. The size of crystallites (d_XRD_) calculated with Equation (1) was 26 ± 8 nm.

### 3.2. Characterization of NCs

In Figure 3, XRD patterns for composites samples are presented. In all samples, diffraction peaks allocated in 30–70° 2θ-range and attributed to spinel ferrite CoFe_2_O_4_ and Zn_0.25_Co_0.75_Fe_2_O_4_ are indexed. The intensity of diffraction peaks is reduced compared to the pure powder sample (Figure S1a) due to high polymer content in the samples. In the low field region allocated diffraction peaks related to the PVDF and PVDF-TrFE polymers. The higher relative intensity of diffraction peak at ~20° in PVDF-TrFE than in PVDF indicates the higher crystallinity of PVDF-TrFE. Oriented and random nanocomposites showed similar XRD patterns (Figure S4a). In 3-component NCs there are three distinguished phases attributed to β-phase of PVDF-TrFE polymer, perovskite structure of BTO, and spinel structure of CFO particles (Figure S4b). The pattern for ZCFO/PVDF-TrFE is similar to the patterns for CFO-based NCs (Figure S4c).

Patterns for PVDF are almost identical to the reported ones in [53]. Where the major phase was the monoclinic α-phase crystal, confirmed by two intensive diffraction peaks at 18.4° and 20.0° and a low intense peak at 26.6°, corresponding to (020), (110), and (021) reflections. Characteristic diffraction peak at 20.6° of β-phase is also presented but it is merged with 110 reflections of dominant α-phase. In PVDF-TrFE polymer, β-phase is more pronounced because it is to form in this modification as follows from the literature [32].

**Figure 4:**
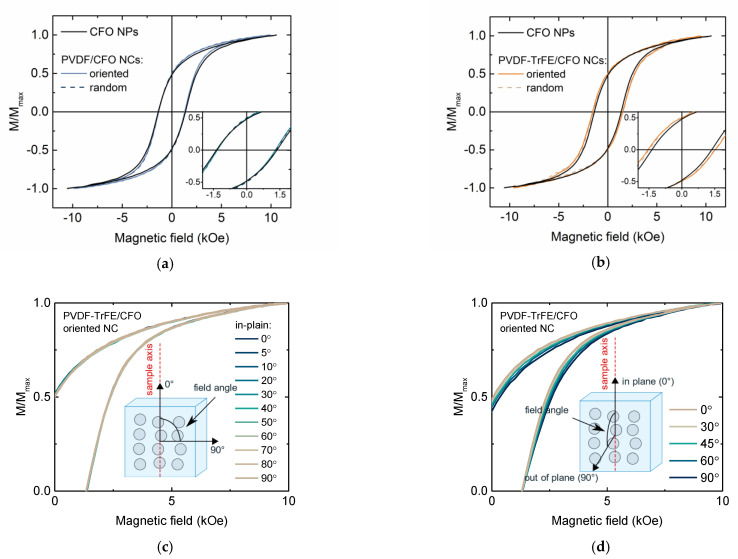
In-plane M-H loops reordered at 300 K for random and ordered (**a**) PVDF/CFO and (**b**) PVDF-TrFE/CFO nanocomposite compared with CFO NPs; M-H loops for ordered PVDF-TrFE/CFO sample as a function of sample axis and field direction in (**c**) in-plane and (**d**) from in-plane to out-of-plane orientations.

Macroscopic magnetic properties of composites samples were studied with VSM at 300 K (Figure 4). M_S_ values of composite samples were reduced concerning CFO powder due to the presence of diamagnetic polymer content. The coercivity field of PVDF-based composites was almost equal to the same value of CFO powder ~1.3 kOe (Figure 4a). Thus matrix stiffness was relatively high, preventing mechanical rotation of particles [54]. PVDF-TrFE-based composites demonstrated a slightly higher coercive field of ~1.5 kOe (Figure 4b). Probably, it is related to slightly lower magnetic interparticle interactions, that were better dispersed in PVDF-TrFE (see explanation below). Interestingly, that the samples ordered in the magnetic field have almost the same magnetic properties as randomly oriented samples. Figure 4c,d shows the angular dependence of M-H loops recorded for oriented CFO/PVDF-TrFE sample in two different orientations of the magnetic field and sample axis (along chains of CFO NPs clusters). In the first case, when the orientation of the sample was always in-plane the hysteresis loops did not depend on the orientation of the field. In the second case, when the direction of the field changed from in-plane to out of plane orientation, a small difference was observed in both random and oriented samples but this was mainly due to the geometrical change of measuring configuration (mutual position of a sample and pick-up coils of VSM).

**Figure 5:**
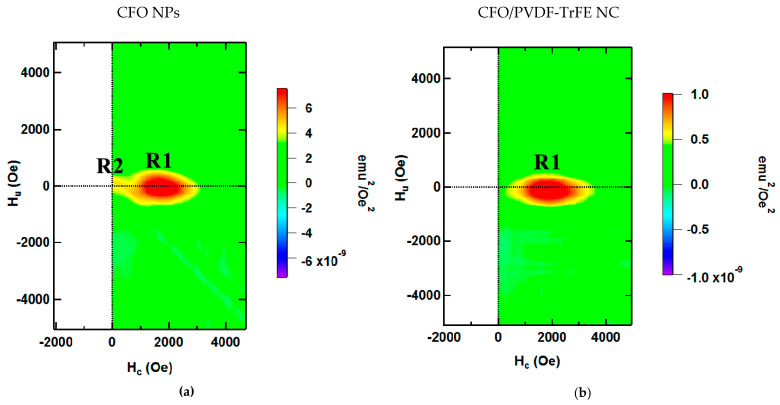
First order reversal curves (FORC) diagram for (**a**) CFO NPs and (**b**) PVDF-TrFE/CFO nanocomposite sample.

Notably, the formation of those ordered chains does not induce any magnetic anisotropy of composite samples. This fact can be explained by the dominant role of intra-aggregate interparticle magnetic interactions on macroscopic magnetic reversal processes. In other words, the arrangement of the clusters’ chains in the magnetic field orients aggregates of several particles but inside those aggregates, the easy axes of magnetic anisotropy of individual NPs are still distributed randomly [54,55]. Individually single-domain magnetic NPs behave according to the Stoner–Wohlfarth model, thus angular dependence of magnetization was expected to change from rectangular to sloped line for easy and hard axes respectively. Indeed, according to TEM investigation (Figure 5b), the produced powder is formed by submicron-size aggregates of densely compacted particles. The dipolar nature of interparticle interactions was confirmed with ΔM-plots (Figure S2b) and FORC diagrams.

The H_r_ profile of FORC diagrams reflects the distribution of coercivities of the particle ensembles (Figure 5a). For close-packed CFO NPs, two main maxima can be observed in the FORC distribution (R1 and R2) [56]. The dominating region R1 reflects the behavior of individual particle clusters. The minor spread in the H_u_ profile indicates that the dipolar magnetic interaction between the particles is dominant: according to ref. [45], magnetic single-domain NPs in clusters is characterized by wider FORC distribution than for individual particles due to a strong and localized interaction. The region R2 results from the interaction among clusters. Indeed, in the case of particles distributed in PVDF-TrFE polymer (Figure 5b), the smaller region R2 is hindered by the sensitivity limit of the VSM device due to the larger distance between clusters [46]; in the case of powder sample (Figure 5a), clusters of NPs are close to each other: two distinct regions, R1 and R2, can be distinguished. R1 regions are identical in Figure 5a,b, it means the dispersion of individual particles in polymer does not affect the assembled clusters: interaggregate interaction has a minor effect, while macroscopic magnetic properties of the samples are determined mainly by their magnetocrystalline anisotropy and interactions in their assemblies (clusters of several NPs). For elastomers in presence of magnetic field during evaporation and without it (ordered and random samples, respectively), FORC-analysis does not show the differences in H_c_ − H_u_ planes. This observation together with results of angular M-H measurements (Figure 4c,d) indicates that even in the case of ordered clusters of NPs, samples are magnetically isotropic; distribution of individual easy axis of particles is random.

**Figure 6:**
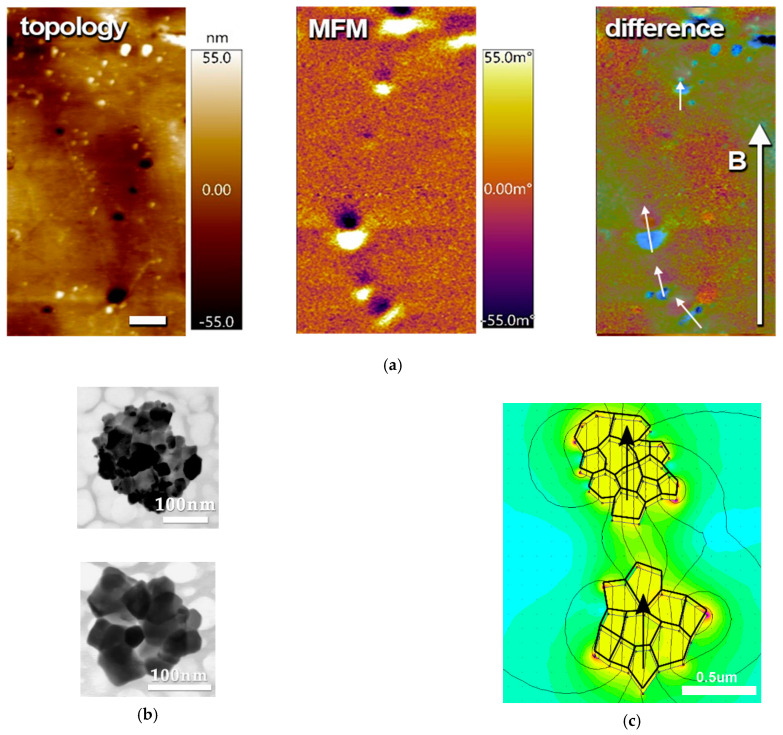
(**a**) Magnetic force microscopy (MFM) images of PVDF-TrFE/CFO nanocomposite: topology, MFM signals, and their difference. Arrow B indicates the direction of the applied magnetic field during polymerization. Scale bar is 2 μm; (**b**) TEM image of separated aggregates of powder CFO NPs; (**c**) illustration of a possible configuration of two aggregates and simulated magnetic field distribution for this configuration.

The micromagnetic structure of prepared composite samples was studied using MFM in zero external magnetic fields (Figure 6). MFM images show magnetically active regions of about 0.1–1 μm formed by NPs aggregates (clusters of several particles) magnetized in the same direction and arranged in chains [19]. The formation of those chains is caused by the applied external magnetic field during polymerization. The contrast spots render magnetized regions that do not match the signal of MFM magnitude with the topology. The appearance of the magnetized regions on topologically flat areas confirms that particles were immersed into the polymer and not exposed on the surface. In composites, evaporated in the absence of a magnetic field, the magnetic contrast from clusters of magnetic particles shows no preferential orientation of their magnetic moments (Figure S7).

To quantitatively evaluate the impact of two sorts of interactions (intra- and interaggregate) on magnetization state, the finite element method was performed utilizing the FEMM software. To fulfill the simulation, a hypothetical case of two aggregates with sizes close to those estimated from MFM (Figure 6a), the shapes close to observed with TEM (Figure 6b), and measured magnetic properties of CFO powder was reproduced (Figure 6c). A situation of collinearly magnetized aggregates (all magnetic moments of individual particles formed those aggregates aligned in a head-to-tail manner) is rendered in Figure 6c. Magnetostatics energy of this configuration was minimal among other considered cases (see more data in Figure S8). For example, configuration with the head-to-head magnetization of aggregates has the maximal energy of interaggregate interactions with a total energy of about 40% higher than in the previous configuration. If one or both aggregates have a close structure with minor stray field and negligible interaggregate interactions, the total energy increased by one and two orders of magnitude respectively. This finding confirms that despite the appearance of magnetic microstructure observed with MFM, its impact on macroscopic magnetic properties is still minor, while magnetic interactions inside aggregates and particles themselves are dominant [57].

### 3.3. Random and Oriented NCs Based on CFO NPs in PVDF and PVDF-TrFE NCs

**Figure 7:**
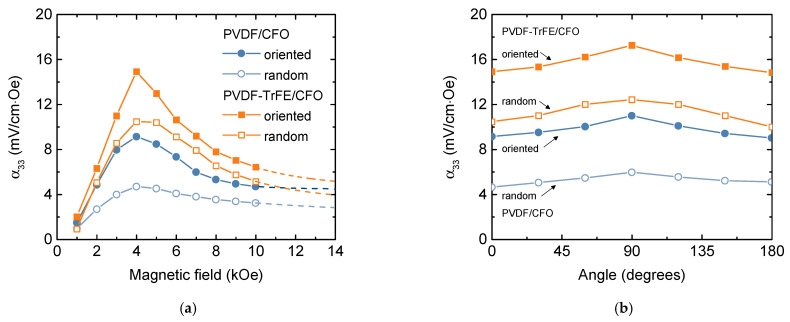
(**a**) Field and (**b**) angular dependencies of the ME voltage coefficient (α_ME_) on DC bias magnetic field for ordered and random PVDF/CFO and PVDFTrFE/CFO composites at AC field frequency of 10 kHz.

The dependence of ME voltage coefficients (α_ME_) versus DC magnetic field (H_DC_) for all composites has a peak-like behavior (Figure 7). The non-monotonous ME response with a maximum at ~4 kOe is related to the magnetization processes of the CFO nanoparticles (see Section 3.5). All measurements were performed at a fixed frequency of 10 kHz, which is below resonant frequency for the samples. The lower frequency was chosen because of the limitations of further biological experiments (long-time treatment at higher frequencies will induce unwanted heating of the system).

The ME voltage coefficients α_ME_ depend on the type of piezoelectric matrix: samples with PVDF-TrFE piezoelectric matrix demonstrates larger ME coefficient in comparison with PVDF-based (Figure 7a), which can be associated with better piezoelectric properties of PVDF-TrFE polymer (d_33_ = −38 pC/N) in comparison with PVDF (d_33_ = −25.8 pC/N) [58]. Observed values of ME coefficients are in the range of reported data on ME polymer composites [15]. However, for an accurate comparison of ME effect, such factors as types of magnetic and ferroelectric components, their magnetic, ferroelectric, piezomagnetic and piezoelectric properties, phase coexistence, mechanical coupling, fabrication techniques should be taken into account. For example, P. Martins et al. observed linear response of ME coefficient versus biasing DC magnetic field in the field up to 5 kOe in Ni_0.5_Zn_0.5_Fe_2_O_4_/PVDF-TrFE nanocomposites [17]. They observed a maximal α_33_ value of 1.35 mV/cm·Oe for composite with 15 wt% Ni_0.5_Zn_0.5_Fe_2_O_4_ nanoparticles (NPs) in 40 kHz AC field with the amplitude of about 1 Oe and DC biasing field of 5 kOe. J. Zhang et al. [59] found a higher α_33_ value of about 40 mV/cm·Oe in CoFe_2_O_4_/PVDF-TrFE measured in the same field condition. In later work, P. Martins et al. compared the values of ME coefficient of X^2+^Fe^3+^_2_O_4_/PVDF-TrFE, where X^2+^ = Zn/Mn, Co, and Fe [18]. The higher value of ME coefficient was observed in nanocomposite with CoFe_2_O_4_ NPs of about 15 nm prepared via the hydrothermal route. Those particles had a coercivity of ~2.5 kOe and magnetization of ~61 emu/g at 10 kOe while the other studied particles exhibit superparamagnetic behavior at room temperature. Measured values of α_ME_ coefficient are lower than in some cases listed above (~17 and ~11 mV/cm·Oe for PVDF-TrFE/CFO and PVDF/CFO samples, respectively), the further improvement can be achieved by the optimization of concentration, composition, and better dispersion of magnetic particles.

The orientation of CFO particles during polymerization enhanced α_ME_ coefficient (~50% for PVDF/CFO and ~30% for PVDF-TrFE/CFO polymer composites). Interesting to note, that even if samples have isotropic magnetic properties, they demonstrated anisotropy magnetoelectric properties. This fact is due to geometrical features of samples: in the in-plane orientation of the field it is more difficult to deform the sample, while the out-of-plane orientation when rectangular sample placed perpendicular to the field direction, is easier. In cases of oriented samples, the angular dependence of the magnetoelectric coefficient becomes slightly sharper (α_ME_(0°)/α_ME_(90°) is ~77% for random and ~83% for oriented PVDF/CFO, ~84% for random and ~86% for oriented PVDF-TrFE/CFO samples).

**Figure 8:**
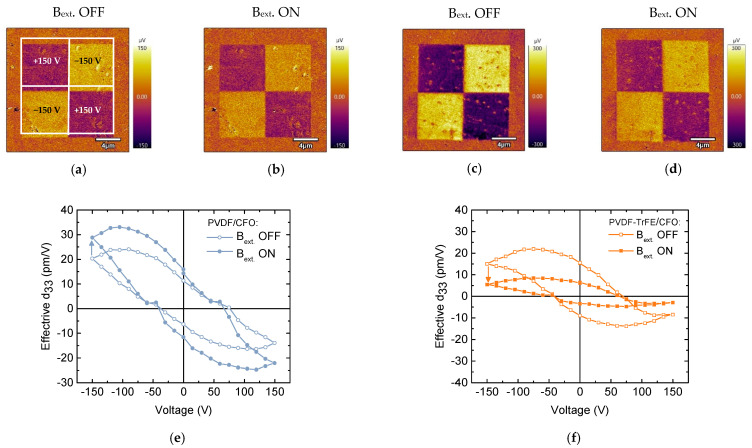
Piezoresponse force microscopy (PFM) images (**a**–**d**) and remnant local piezoelectric hysteresis loops (**e**,**f**) at applied (B_ext._ ON) and switched off (B_ext._ OFF) the magnetic field (B_ext._ = 1.4 kOe) for (**a**,**b**,**e**) PVDF/CFO and (**c**,**d**,**f**) PVDF-TrFE/CFO samples.

To study the piezoelectrical domain switching behavior of the PVDF/CFO and PVDF-TrFE/CFO samples, the “mini” chessboard structures were written without a magnetic field (Figure 8a,c). The dark and bright square areas (7.5 × 7.5 µm^2^) correspond to applied +150 V and −150 V DC biases, respectively. Strong PFM contrast confirms the complete switching process in composite polymer samples under poling. The domains created are rather stable in time. It was found that the magnitude of the PFM signal of polarized regions for sample PVDF-TrFE/CFO is 4 times higher than for PVDF/CFO sample. Then the samples were placed in a magnetic field (B_ext._ = 1.4 kOe) and the same polarized area was scanned again (Figure 8b,d). It is experimentally shown that for PVDF/CFO sample, an external magnetic field increases (~30%) the signal of the remnant piezoelectric response or effective d_33_. For comparison, the PVDF-TrFE/CFO sample shows a ~64% reduction in the effective d_33_. Besides, the values of the effective piezoelectric coefficient d_33_ (Figure 8e,f) for PVDF/CFO composite are higher than for PVDF-TrFE/CFO. These experiments demonstrate locally induced ME coupling on composites, which is associated with the deformation of the piezoelectric matrix by magnetic CFO NPs. Thus, this method additionally confirms the magnetoelectric nature of the composites [60].

### 3.4. Further Improvements of ME Efficiency

**Figure 9:**
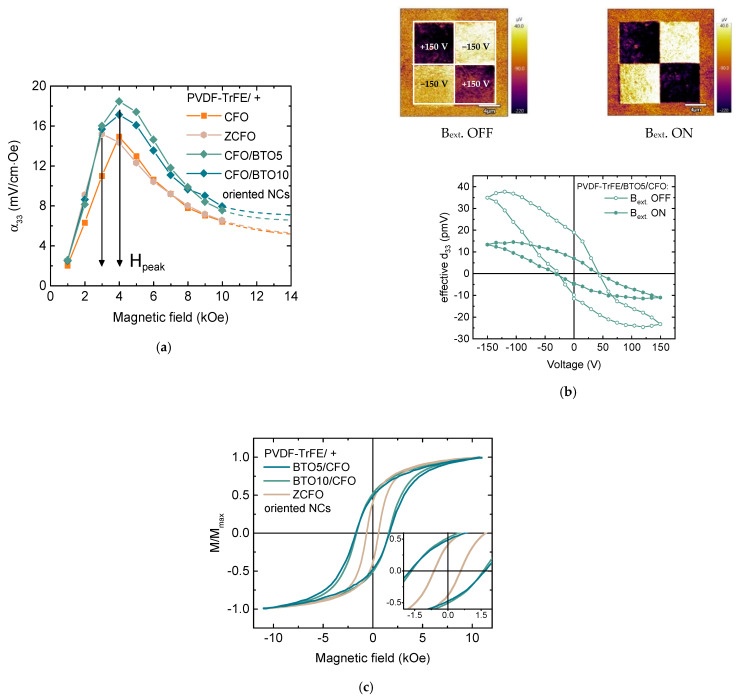
(**a**) Dependencies of the ME voltage coefficient (α_ME_) on DC bias magnetic field composites at AC field frequency of 10 kHz for PVDF-TrFE -based samples with ZCFO, BTO5/CFO and BTO10/CFO fillers (PVDF-TrFE/CFO is shown again for comparison); (**b**) piezoresponse force microscopy (PFM) images and remnant local piezoelectric hysteresis loops at applied and switched off the magnetic field (B_ext._ = 1.4 kOe) for PVDF-TrFE/BTO5/CFO sample; (**c**) M-H loops at 300 K for PVDF-TrFE/ZCFO, PVDF-TrFE/BTO5/CFO and PVDF-TrFE/BTO10/CFO NCs.

Further improvement of ME efficiency was achieved via the inclusion of one additional component, with 5% and 10% weight content of piezoelectric BaTiO_3_ (BTO) particles in more efficient ordered PVDF-TrFE/CFO NC. Thus, 3-component NC samples, ordered PVDF-TrFE/BTO5/CFO and PVDF-TrFE/BTO10/CFO, were obtained. The adding of BTO fillers led to a small enhancement in the ME effect (Figure 9a), which is related to the contribution of BTO with higher piezoresponse in comparison with PVDF-based polymers (d_33_ = 191 pC/N [61]). When the concentration of BTO increased from 5 to 10% the ME voltage coefficients α_ME_ decreased from 18.5 to 17 mV/cm·Oe. That was attributed to a reduced quality of the crystallization of polymer when it is overfilled. Nonetheless, those values are sensory higher than this value for ordered PVDF-TrFE/CFO NC. The local piezoelectric hysteresis loop measured utilizing PFM demonstrates the response of d_33_ BTO and PVDF-TrFE components to the applied magnetic field as a result of magnetoelectric interaction (Figure 9b). The introduction of diamagnetic BTO particles into composites does not affect their magnetic properties and they are almost identical within experimental error with 2-component PVDF-TrFE/CFO NC.

Additionally, the Zn substituted cobalt ferrite (Zn_0.25_Co_0.75_Fe_2_O_4_, ZCFO) NPs with the same size as CFO NPs, the slightly higher saturation magnetization, and lower magnetic anisotropy were used to tune a magnetoelectric response. M-H loop of PVDF-TrFE/ZCFO is shown in Figure 9c. PVDF-TrFE/ZCFO NC has much lower coercivity (H_C_~0.6 kOe) and irreversibility fields (H_irr_~3.8 kOe) than those for the family of PVDF-TrFE/CFO NCs (H_C_~1.5 kOe; H_irr_~7 kOe). NC with ZCFO particles shows slightly higher ME performance, which more likely can be attributed to the higher M_S_ value of ZCFO NPs. The higher value of M_S_ leads to the stronger magnetostatic interactions and thus to the stronger interactions of particle clusters. A more significant change was detected in the position of peak (H_peak_) in the field dependence of the ME voltage coefficients α_ME_. The H_peak_ was reduced at a factor 0.56–0.71, which is quite close to the ratio of magnetic anisotropy constants K_ZCFO_/K_CFO_~0.6.

### 3.5. The Origin of ME Effect

**Figure 10:**
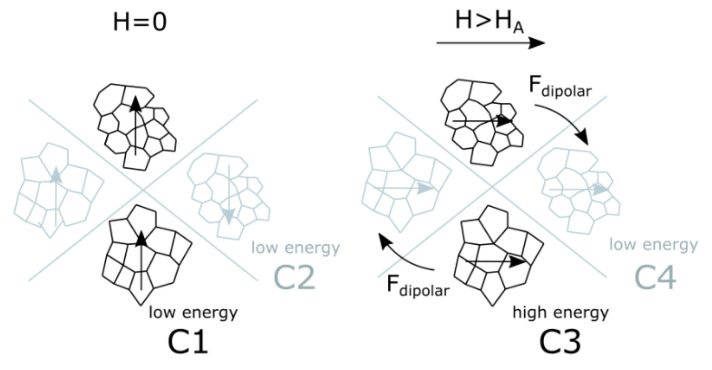
Schematic diagram explaining the mechanism of arising of dipolar interactions leading to the displacement of aggregates of CFO NPs: in zero magnetic fields (explanation is in the text).

The magnetoelectric effect was explained through magnetostatic interactions. In a zero magnetic field in an equilibrium state, magnetic moments of aggregates are parallel (C1) or antiparallel (C2) depending on the initial location (Figure 10, symmetrical cases are not shown). When an external magnetic field higher than the magnetic anisotropy of particles is applied (H > H_A_), a high-energy configuration (C3) is formed because of magnetization of particles. In this case, dipolar forces will invoke a rearrangement of particles to reach another low-energy configuration (C4) if the viscosity of the matrix will allow (as in liquid precursor of polymer in Figure 1b, when chains were formed) or to arising of mechanical stresses if the matrix is rigid [54,55]. In the case of 3-component NCs, the polymer matrix transfers the mechanical stresses from matrix to BTO particles by elastic coupling that causes their electric polarization due to piezoelectric effect. This model can explain the reason for the increase of magnetoelectric response in oriented samples. Indeed, in samples with the random distribution of magnetic particles, configurations C1 and C2 have the same probability, but the low-energy C2 case in an external magnetic field will form just another low-energy case C4. Thus only 50% of particles after a magnetic field is ON will be in the high-energy configuration generating stronger mechanical stresses on the piezopolymer matrix and causing a stronger magnetoelectric response. This model also can explain, why the field-dependence of magnetoelectric response has peak-like behavior. Reaching a certain field close to the magnetic anisotropy field of CFO particles (H_A_), almost all local magnetic moments of magnetic particles are oriented along the external magnetic field. For the case of ZCFO NPs with a lower anisotropy field (H_A_ for single-domain magnetic particles is proportional to H_irr_ [34]), the value of H_peak_ is proportionally lower. Further increase of the magnetic field only slightly rotates those moments in direction of the magnetic field but, at the same time, the external magnetic field hides the gradient of the local field. The magnetic force acting on each magnetic dipole (in this contest, an aggregate) is proportional to the multiplication of the value of magnetic moment on the gradient of the magnetic field (M·grad(B)) formed by external field and neighbor sources of the magnetic field (such as neighbor aggregates). Thus, the reduction of the gradient of the magnetic field will lead to the reduction of energy of interparticle (or interaggregate) interactions and reduce the strain of the piezoelectric matrix.

### 3.6. Biological Tests

**Figure 11:**
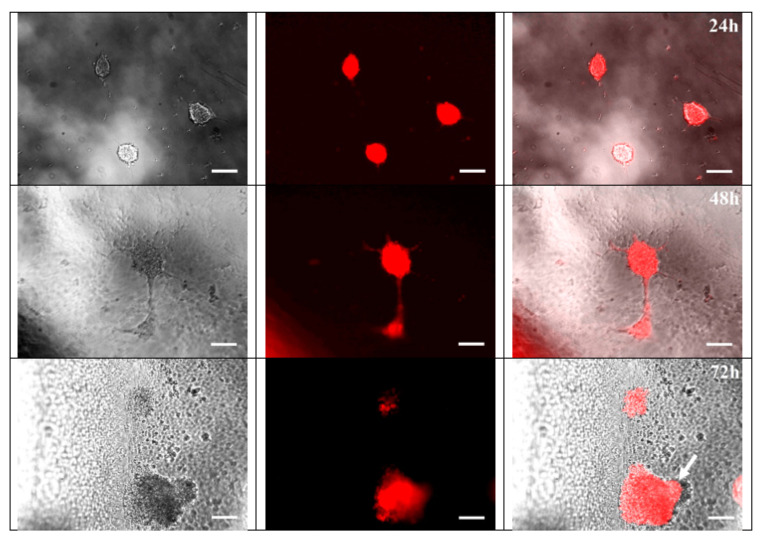
Images of neural crest stem cells (bNCSC), expressing red fluorescence signal (RFP) after 24 h, 48 h, 72 h of culture on PVDF substrate (×20); arrow indicates cells migrating from the neurosphere. Scale bar is 25 μm.

Previously it has been shown that PVDF can be used for attachment and differentiation of cells from different tissues: cardiovascular [26], osteogenic [25], muscle [62], and neuronal cells [63]. The neural crest stem cells (bNCSC) adhesion, survival, and differentiation were compatible with the PVDF material in line with previous findings on hippocampal neurospheres [27]. However, the effect of electromagnetic activities on neurogenesis remains controversial. The effect of PVDF on neural stem cells isolated in the later time of embryonic development was shown previously [63]. However, isolation of cells at an earlier stage of embryonic development gives the ability to direct their differentiation is more addressed, but biocompatibility of materials should be studied additionally. To test the biocompatibility of PVDF, we used bNCSC that reliably demonstrated their ability to generate neurons and glial cells in vitro [64,65]. This type of cells is characterized by highly proliferative activity (approximate cell population doubling time ~15–18 h) and was isolated at the early stage of embryonic development (11.5 days mouse embryos). After 24 h of bNCSCs culture on PVDF substrate, the number of cells was increased, with some of them attached to the polymer and forming neurospheres, as evidence of strong proliferation activity (Figure 11). After 48 h the size of neurospheres was markedly increased. In addition, a small number of elongated bNCSCs were present, which suggested the onset of migrated and differentiated cells (Figure 11). After 72 h, the processes of cell migration from neurospheres on the polymer substrate became more prominent. This process resulted in a reduced density of intercellular connections and possibly due to increased adhesion to the surface of the substrate. bNCSC placed on the material strongly attached and differentiated during 3 days, which is in agreement with the previously published protocol [66,67].

**Figure 12:**
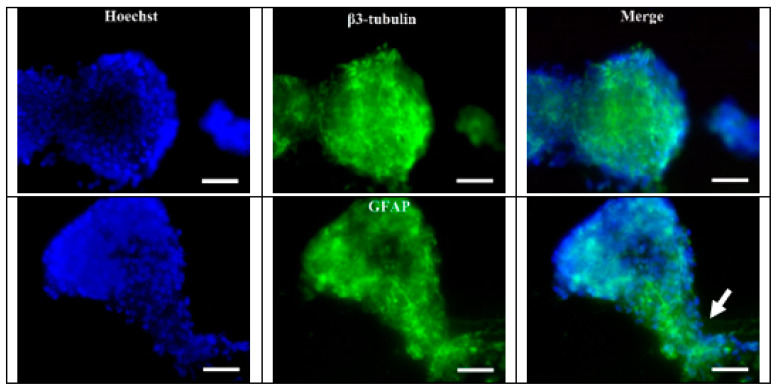
βIII-tubulin and GFAP (green) expression bNCSCs growing on PVDF substrate (×40). Blue–Hoechst nuclear stain; arrow indicates cells migrating from the neurosphere. Scale bar = 25 μm.

Two markers of cell differentiation were used for the immunofluorescent staining procedure: neuronal—β3-tubulin and glial—GFAP (glial fibrillary acidic protein). Both β3-tubulin positive cells and GFAP positive cells were present in the stained samples (Figure 12). These data can be interpreted as the cells retaining their potential for differentiation into neurons and glial cells in PVDF substrate. In addition, the stained samples showed traces of the migratory activity of bNCSC cells on the PVDF surface (Figure 12).

Neuronal stem cells isolated at the early embryonic stage cultivated on PVDF-based surface were able to proliferate and differentiate into main types of neural cells (neurons and glial cells). This finding together with an expected possibility to stimulate and modulate those processes remotely via magnetic field invoking local electric polarization of interface suggests that the developed materials can be used as bio-interfaces. For example, as a tool for neuronal stem cell cultivation and targeted deafferentation for future application in the treatment of neurodegenerative disorders and spinal cord injury and brain damage.

## 4. Conclusions

We have prepared and studied a set of samples of polymer-based nanocomposites having magnetic and magnetoelectric properties owing to the inclusion of magnetic nanoparticles in piezopolymers. New strategies to increase the magnetoelectric performance of PVDF- and PVDF-TrFE-based nanocomposites by the orientation of NPs clusters in chains in the polymer matrix and by the creation of 3-component nanocomposites by adding one additional component (ferroelectric particles) have been demonstrated. In our study, the magnetoelectric voltage coefficient (α_ME_) of oriented 3-component PVDF-TrFE/BTO5/CFO composites was about 18.5 mV/cm·Oe that is four times higher than it is in the randomly oriented 2-component PVDF/CFO composite. A model based on magnetostatic interactions of clusters of magnetic nanoparticles with randomly distributed easy axes for the explanation of the ME transformation in 3-component composites has been suggested. Local magnetic and piezoelectric properties have been studied employing scanning probe microscopy. Further researches will be aimed to increase the magnetoelectric performance by changing particle (both magnetic and ferroelectric) size, shape, and concentration in such composites. Currently, the work on the use of obtained magnetoelectric composites as a bioactive interface is in progress. The possibility to remotely vary the surface charge by applying the magnetic field can be used to modulate the live process of neuronal stem cells, e.g., to control and induce their differentiation. Our results suggest that PVDF-based substrates are biocompatible for neuronal stem cells isolated at the early embryonic stage and thus they can be used as a matrix for cell cultivation for future application in the treatment of neurodegenerative disorders and spinal cord injury and brain damage. Furthermore, the functionalization of the PVDF substrate may contribute to the guidance of stem cell differentiation toward the required type of cells and may be used for the development of new in vitro differentiation strategies.

## Data Availability

The data presented in this study are available on request from the corresponding author.

## Supplementary Materials

The following are available online at https://www.mdpi.com/article/10.3390/nano11051154/s1. Figure S1: (a) XRD pattern of CoFe_2_O_4_ and Zn_0.25_Co_0.75_Fe_2_O_4_ NPs; (b) TEM micrograph and size distribution of CoFe_2_O_4_ NPs. Figure S2: (a) Room temperature (~300 K) M-H loop (b) ΔM-plot of CoFe_2_O_4_ and Zn_0.25_Co_0.75_Fe_2_O_4_ NPs in form of powder. Figure S3: XRD pattern of BaTiO_3_ particles. Figure S4: Comparison of XRD patterns for (a) random and oriented CFO/PVDF and CFO/PVDF-TrFE nanocomposites; (b) nanocomposites with 5 and 10% of BaTiO_3_ particles; (c) ZCFO/PVDF-TrFE nanocomposite. Figure S5: Optical images of aligned clusters of CFO NPs in (a) PVDF-TrFE and (b) PVDF polymers. Figure S6: Atomic force microscopy (AFM) images of (a) PVDF-TrFE/CFO and (b) PVDF/CFO NCs; histograms of the pore depth distribution obtained using the WSxM program for (c) PVDF-TrFE/CFO and (d) PVDF/CFO NCs. The median value and standard deviation (σ) of log-normal distribution are presented. Figure S7: Magnetic force microscopy (MFM) images of PVDF-TrFE/CFO nanocomposite evaporated in the absence of magnetic field: (a) topology and (b) MFM signals. Figure S8: Results of FEMM simulation of magnetic induction (B) for different configurations of initial magnetization of particles aggregates: (a) “head-to-tail” magnetization state; (b) “head-to-head” magnetization state; (c) one aggregate has a close structure and the second is uniformly magnetized; (d) two aggregates have close structures.
