# Supplementary material for: Boosting Magnetoelectric Effect in Polymer-Based Nanocomposites
Source: Nanomaterials (Basel). 2021 Apr 28;11(5):1154. doi: 10.3390/nano11051154 (PMC8146360; doi:10.3390/nano11051154)
Supplement: Supplementary file 1 [file nanomaterials-11-01154-s001.zip › nanomaterials-1180505-supplementary.pdf]

Article

Boosting Magnetoelectric Effect in Polymer-Based Nanocomposites

Alexander Omelyanchik [1,2], Valentina Antipova [1], Christina Gritsenko [1], Valeria Kolesnikova [1], Dmitry Murzin [1], Yilin Han [3], Andrei V. Turutin [4,5], Ilya V. Kubasov [4], Alexander M. Kislyuk [4], Tatiana S. Ilina [4], Dmitry A. Kiselev [4], Marina I. Voronova [4], Mikhail D. Malinkovich [4], Yuriy N. Parkhomenko [4], Maxim Silibin [6,7,8], Elena N. Kozlova [3], Davide Peddis [2,9], Kateryna Levada [1], Liudmila Makarova [1,10], Abdulkarim Amirov [1,11,*] and Valeria Rodionova [1,*]

1. REC Smart Materials and Biomedical Applications, Immanuel Kant Baltic Federal University, 236041 Kaliningrad, Russia; asomelyanchik@kantiana.ru (A.O.); vantipova1@kantiana.ru (V.A.); christina.byrka@gmail.com (C.G.); vgkolesnikova1@kantiana.ru (V.K.); dvmurzin@yandex.ru (D.M.); elevada@kantiana.ru (K.L.); la.loginova@physics.msu.ru (L.M.)
2. Department of Chemistry and Industrial Chemistry (DCIC), University of Genova, 16146 Genova, Italy; davide.peddis@unige.it
3. Biomedical Centre, Department of Neuroscience, Uppsala University, 751 24 Uppsala, Sweden; yilin.han@neuro.uu.se (Y.H.); elena.kozlova@neuro.uu.se (E.N.K.)
4. Laboratory of Physics of Oxide Ferroelectrics and Department of Materials Science of Semiconductors and Dielectrics, National University of Science and Technology MISiS, 119049 Moscow, Russia; aturutin92@gmail.com (A.V.T.); kubasov.ilya@gmail.com (I.V.K.); akislyuk94@gmail.com (A.M.K.); ilina.tatina@gmail.com (T.S.I.); dm.kiselev@gmail.com (D.A.K.); mvoron@bk.ru (M.I.V.); malinkovich@yandex.ru (M.D.M.); parkh@rambler.ru (Y.N.P.)
5. Department of Physics and I3N, University of Aveiro, 3810-193 Aveiro, Portugal
6. Institute of Advanced Materials and Technologies, National Research University of Electronic Technology "MIET", 124498 Moscow, Russia; sil_m@mail.ru
7. Institute for Bionic Technologies and Engineering, I.M. Sechenov First Moscow State Medical University, 119991 Moscow, Russia
8. Scientific-Manufacturing Complex "Technological Centre" Shokin Square, House 1, Bld. 7, Zelenograd, 124498 Moscow, Russia
9. Institute of Structure of Matter–CNR, Monterotondo Stazione, 00016 Rome, Italy
10. Faculty of Physics, Lomonosov Moscow State University, 1-2 Leninskie Gory, 119234 Moscow, Russia
11. Amirkhanov Institute of Physics of Dagestan Federal Research Center, Russian Academy of Sciences, Makhachkala 367003, Russia

* Correspondence: amiroff_a@mail.ru (A.A.); vvrodionova@kantiana.ru (V.R.)

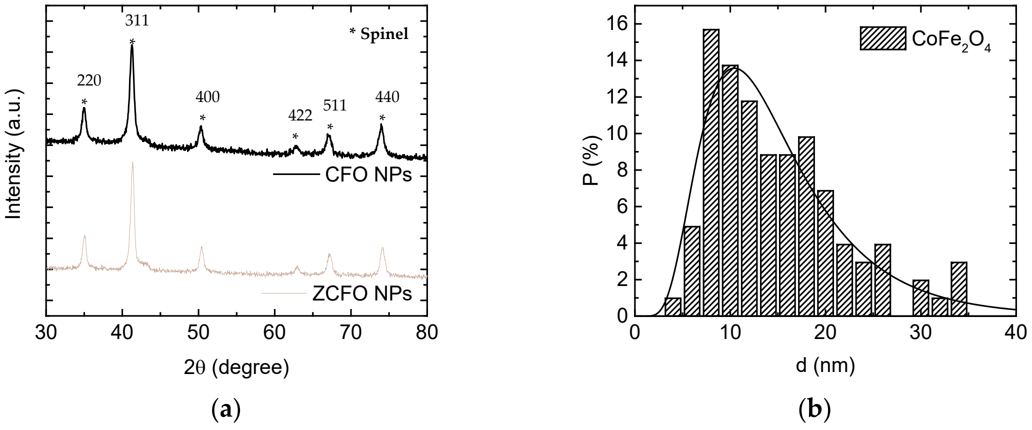

Figure S1. (**a**) XRD pattern of $CoFe_2O_4$ and $Zn_{0.25}Co_{0.75}Fe_2O_4$ NPs; (**b**) TEM micrograph and size distribution of $CoFe_2O_4$ NPs.

Magnetic interparticle interactions were investigated by measuring the isothermal remanent magnetization (IRM) and the direct current demagnetization (DCD) [1,2].

M_{IRM}(H) was measured on the demagnetized sample applying a set of increasing fields (up to 10 kOe), removing them and recording the remanent magnetization after each iteration. M_{DCD}(H) was measured saturating first the sample, applying a set of increasing reverse fields and recording the remanent magnetization after each iteration. According to the Stoner–Wohlfarth model for an assembly of non-interacting randomly oriented MNPs with uniaxial anisotropy the two remanent magnetization curves are correlated by the following equation: $M_{DCD}(H) = 1 - 2 \cdot M_{IRM}(H)$. The effect of interparticle interactions can be evaluated by the Kelly equation [3]:

$$\Delta M(\mathrm{H}) = M_{DCD}(\mathrm{H}) - 1 + 2 \cdot M_{IRM}(H) \tag{1}$$

where ΔM allows one to estimate the level of interactions. ΔM = 0 for an ideal situation with the absence of interactions. A negative value indicates the predominance of dipolar interactions, whereas a positive value indicates the predominance of exchange interactions. CFO powder sample shows a negative deviation with the intensity of about ~0.1 that suggests that in powder sample the interparticle dipolar interactions are dominant.

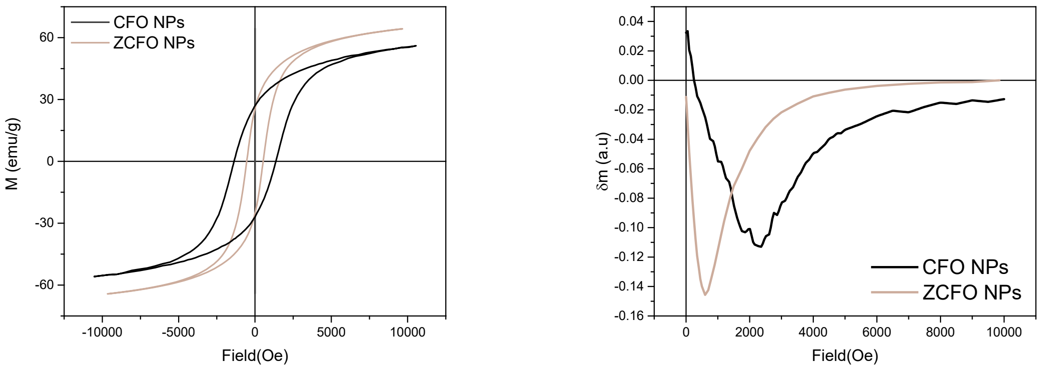

(**a**) (**b**)

Figure S2. (**a**) Room temperature (~300 K) M-H loop (**b**) ΔM-plot of $CoFe_2O_4$ and $Zn_{0.25}Co_{0.75}Fe_2O_4$ NPs in form of powder.

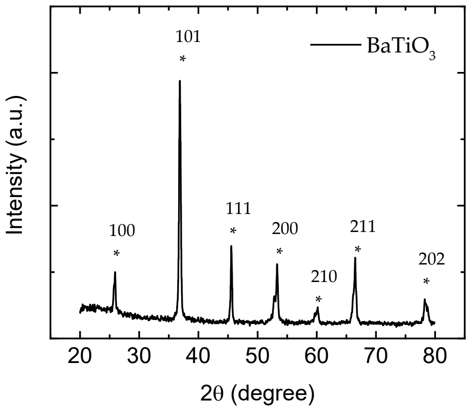

Figure S3. XRD pattern of $BaTiO_3$ particles.

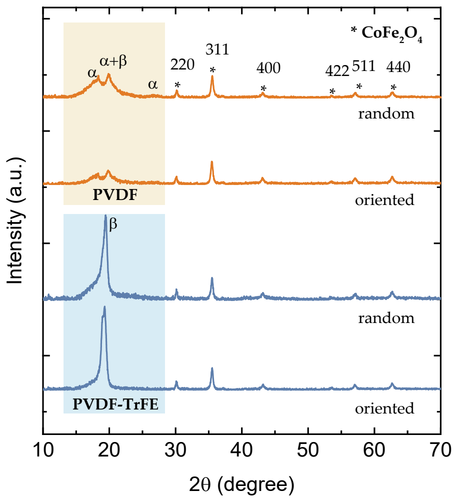

(a)

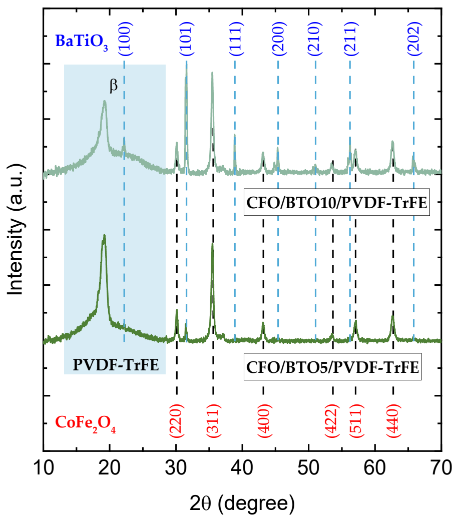

(b)

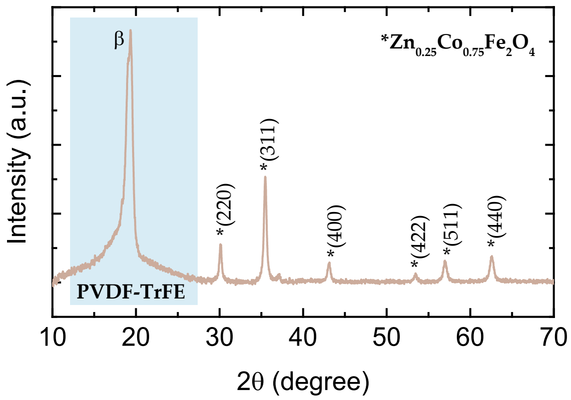

(c)

Figure S4. Comparison of XRD patterns for (**a**) random and oriented CFO/PVDF and CFO/PVDF-TrFE nanocomposites; (**b**) nanocomposites with 5 and 10% of $BaTiO_3$ particles; **(c)** ZCFO/PVDF-TrFE nanocomposite.

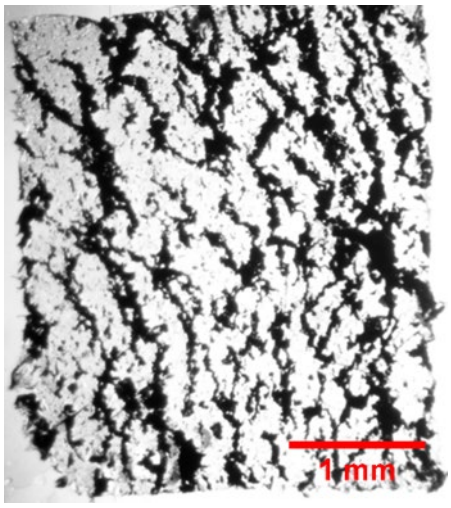

(a)

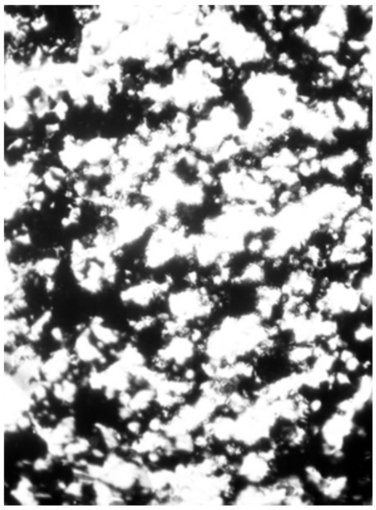

(b)

Figure S5. Optical images of aligned clusters of CFO NPs in **(a)** PVDF-TrFE and **(b)** PVDF polymers.

The pore depth was estimated using the WSxM software [4]. Pore depth histograms were plotted using flood functions. By choosing the function "Find holes", it is possible to display all points below a certain height. By default WSxM sets flooding images by using the

topography image and showing holes in different colours and holes with a height larger than the one selected here will neither be displayed nor considered during calculus. Next, a function was used to create a histogram of height. The maximum height histogram shows a histogram of the maximum value of the difference between the cutoff height and the point's height.

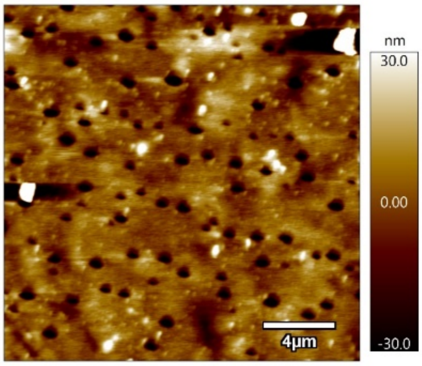

(a) Rms = 21 nm

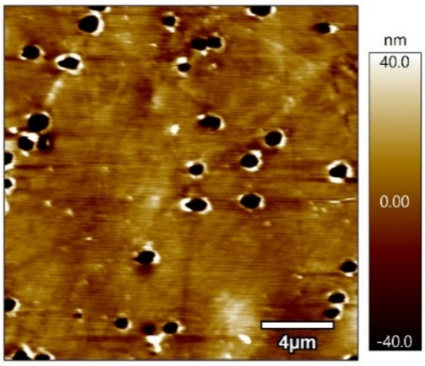

(b) Rms = 33 nm

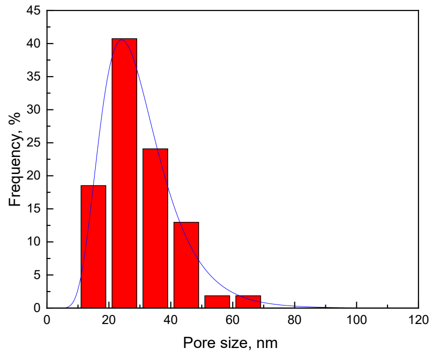

(c) Pore size (median) = 30 nm; σ = 12

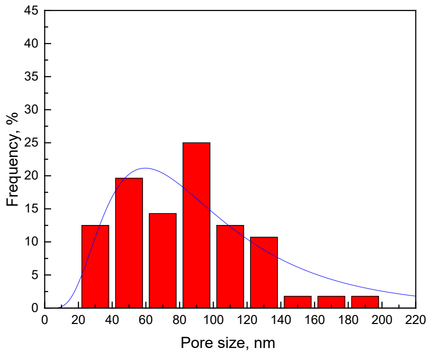

(d) Pore size (median) = 100 nm; σ = 64

Figure S6. Atomic force microscopy (AFM) images of **(a)** PVDF-TrFE/CFO and **(b)** PVDF/CFO NCs; histograms of the pore depth distribution obtained using the WSxM program for **(c)** PVDF-TrFE/CFO and **(d)** PVDF/CFO NCs. The median value and standard deviation (σ) of log-normal distribution are presented.

Topography

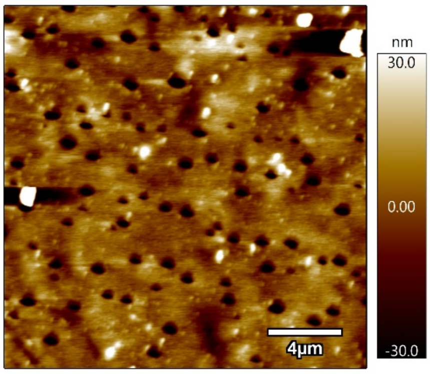

(a)

MFM image

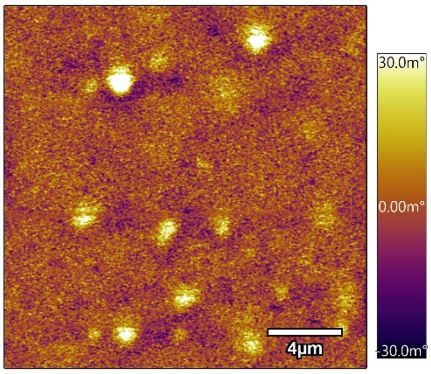

(b)

Figure S7. Magnetic force microscopy (MFM) images of PVDF-TrFE/CFO nanocomposite evaporated in the absence of magnetic field: (**a**) topology and (**b**) MFM signals.

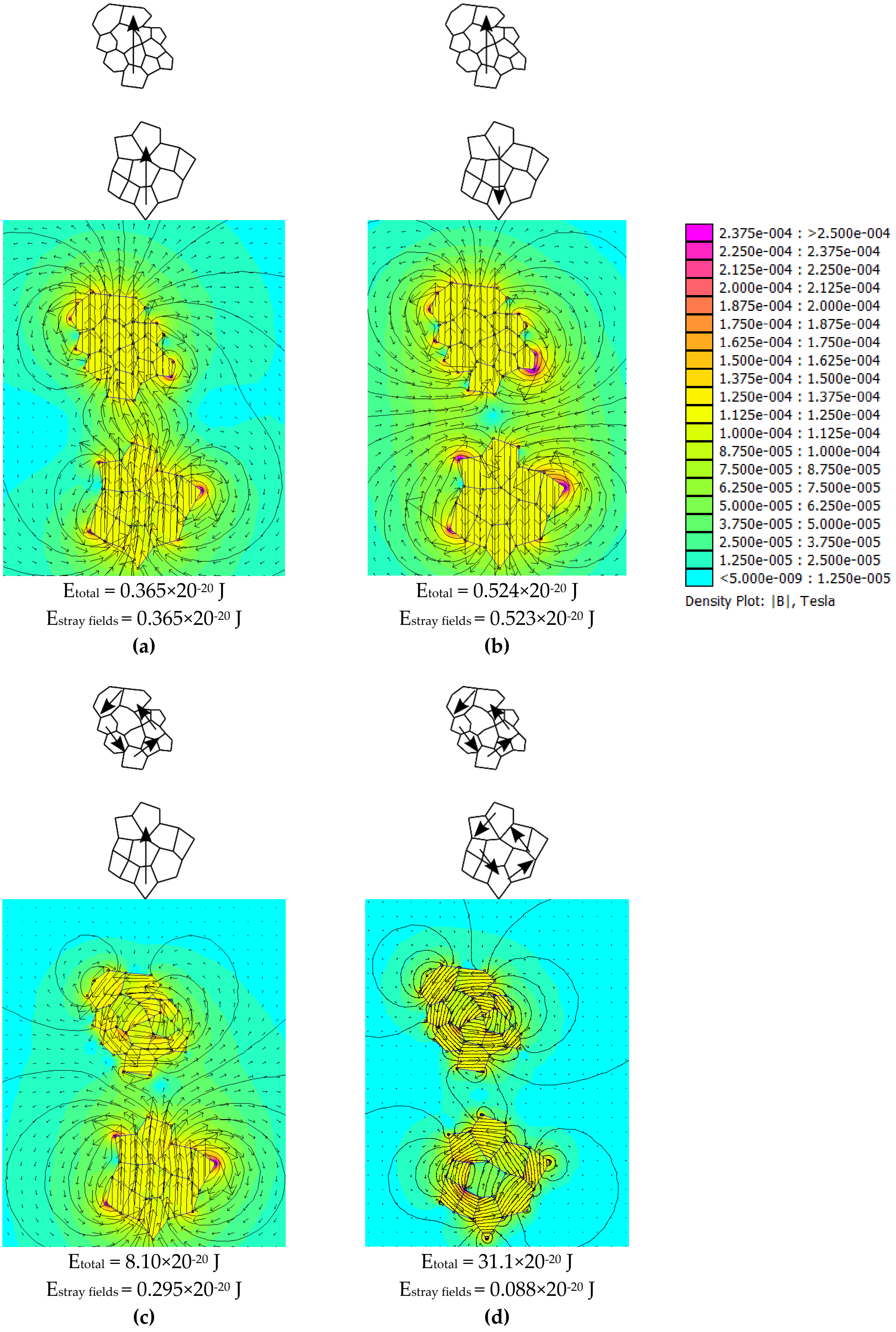

Figure S8. Results of FEMM simulation of magnetic induction (B) for different configurations of initial magnetization of particles aggregates: **(a)** "head-to-tail" magnetization state; **(b)** "head-to-head" magnetization state; **(c)** one aggregate has a close structure and the second is uniformly magnetized; **(d)** two aggregates have close structures.

References

1. Peddis, D.; Jönsson, P.E.; Laureti, S.; Varvaro, G. Magnetic interactions: A tool to modify the magnetic properties of materials based on nanoparticles. In *Frontiers of Nanoscience*; Binns, C., Ed.; Elsevier B.V: Oxford, UK, 2014; Vol. 6, pp. 129–188 ISBN 9780080983530.

2. García-Otero, J.; Porto, M.; Rivas, J. Henkel plots of single-domain ferromagnetic particles. *J. Appl. Phys.* **2000**, *87*, 7376.

3. Kelly, P.E.; O'Grady, K.; Mayo, P.L.; Chantrell, R.W. Switching mechanisms in cobalt-phosphorus thin films. *IEEE Trans. Magn.* **1989**, *25*, 3881–3883.

4. Horcas, I.; Fernández, R.; Gómez-Rodríguez, J.M.; Colchero, J.; Gómez-Herrero, J.; Baro, A.M. WSXM: A software for scanning probe microscopy and a tool for nanotechnology. *Rev. Sci. Instrum.* **2007**.
